# Radiotherapy Alone Versus Concurrent or Adjuvant Chemoradiotherapy for Nasopharyngeal Carcinoma Patients with Negative Epstein–Barr Virus DNA after Induction Chemotherapy

**DOI:** 10.3390/cancers15061689

**Published:** 2023-03-09

**Authors:** Fangfang Kong, Guangsen Pan, Chengrun Du, Chaosu Hu, Hongmei Ying

**Affiliations:** 1 Department of Radiation Oncology, Fudan University Shanghai Cancer Center, Shanghai 200032, China; 2 Department of Oncology, Shanghai Medical College, Fudan University, Shanghai 200032, China; 3 Shanghai Clinical Research Center for Radiation Oncology, Shanghai 200032, China; 4 Shanghai Key Laboratory of Radiation Oncology, Shanghai 200032, China

**Keywords:** nasopharyngeal carcinoma, EBV DNA, induction chemotherapy, concurrent chemotherapy, propensity score-matched analysis

## Abstract

**Simple Summary:**

Induction chemotherapy (IC) plus concurrent chemoradiotherapy has been recommended as the standard treatment for locoregionally advanced nasopharyngeal carcinoma (LA-NPC). However, concurrent chemotherapy was associated with increased toxicities, poor tolerance, and low completion rates. The aim of this study was to compare the efficacy and toxicity of IC + radiotherapy (RT) and IC + concurrent or adjuvant chemoradiotherapy (IC + CCRT/AC) in patients with negative post-IC EBV DNA. The results showed that IC + RT alone displayed similar efficacy to IC + CCRT/AC. The omission of concurrent or adjuvant chemotherapy did not increase locoregional or distant failure. However, patients treated with IC + RT had fewer acute toxicities than those with IC + CCRT/AC. Our finding provided evidence that the omission of concurrent or adjuvant chemotherapy may be feasible for patients with negative EBV DNA after induction chemotherapy.

**Abstract:**

The purpose of this study was to compare the efficacy and toxicity of induction chemotherapy (IC) plus radiotherapy (RT) and IC plus concurrent or adjuvant chemoradiotherapy (CCRT/AC) in nasopharyngeal carcinoma (NPC) patients with negative Epstein–Barr virus DNA (EBV DNA) after IC. A total of 547 NPC patients with negative plasma EBV DNA post-IC were included. Patients were classified into the IC + RT group and the IC + CCRT/AC group. Locoregional relapse-free survival (LRFS), distant metastasis-free survival (DMFS), overall survival (OS), and progression-free survival (PFS) were estimated and compared using the Kaplan–Meier method. Propensity score matching (PSM) was performed to balance the variables. The median follow-up time was 37 months. The 3-year LRFS, DMFS, OS, and PFS rates for the whole group were 92.2%, 92.4%, 96.4%, and 84.4%, respectively. There was no significant difference in LRFS, DMFS, OS, and PFS between the IC + RT and the IC + CCRT/AC groups, both before PSM (3-year rates of 91.1% vs. 92.6%, *p* = 0.94; 95.6% vs. 91.5%, *p* = 0.08; 95.2% vs. 96.8%, *p* = 0.80; 85.9% vs. 84.0%, *p* = 0.38) and after PSM (90.7% vs. 92.7%, *p* = 0.77; 96.8% vs. 93.7%, *p* = 0.29; 94.5% vs. 93.9%, *p* = 0.57; 84.7% vs. 85.6%, *p* = 0.96). Multivariate analysis demonstrated that the treatment schedule was not an independent predictor for survival rates. Patients in the IC + RT group had fewer treatment-related acute toxicities and better tolerance. IC + RT displayed similar survival outcomes as IC + CCRT/AC for NPC patients with negative post-IC EBV DNA.

## 1. Introduction

Nasopharyngeal carcinoma (NPC) is an endemic tumor that is prevalent in southeast Asia, especially in southern China [1]. Induction chemotherapy (IC) combined with concurrent chemoradiotherapy (CCRT) is currently the standard treatment for locoregionally advanced NPC (LA-NPC) [2,3]. However, the evidence of concurrent chemotherapy (CCT) mainly comes from 2D radiotherapy (RT) [4]. In the era of intensity-modulated radiotherapy (IMRT), the value of CCT remains controversial. Moreover, CCT was associated with increased toxicities, which would compromise patients’ compliance and life quality, and increase the risk of treatment-related death [4,5,6,7]. After RT, compliance with adjuvant chemotherapy (AC) was also poor [8,9]. Recently, several retrospective studies have explored the de-intensified treatment of RT alone versus CCRT after IC for LA-NPC, but the results were inconsistent [10,11,12,13]. Due to the heterogeneity of LA-NPC, de-intensified treatment for unselected patients may cause treatment failure.

EBV DNA is an important biomarker of NPC [14]. Many studies have shown that post-IC EBV DNA was an independent predictor of treatment outcome [15,16,17,18]. Undetectable post-IC EBV DNA was associated with superior survival rates. However, there is no report about de-intensified treatment based on post-IC EBV DNA status.

In this study, we aimed to compare the efficacy and toxicity of IC + RT and IC + CCRT/AC in patients with negative post-IC EBV DNA and evaluate the feasibility of omitting concurrent or adjuvant chemotherapy for these low-risk patients.

## 2. Methods and Materials

### 2.1. Patients

**Figure 1:**
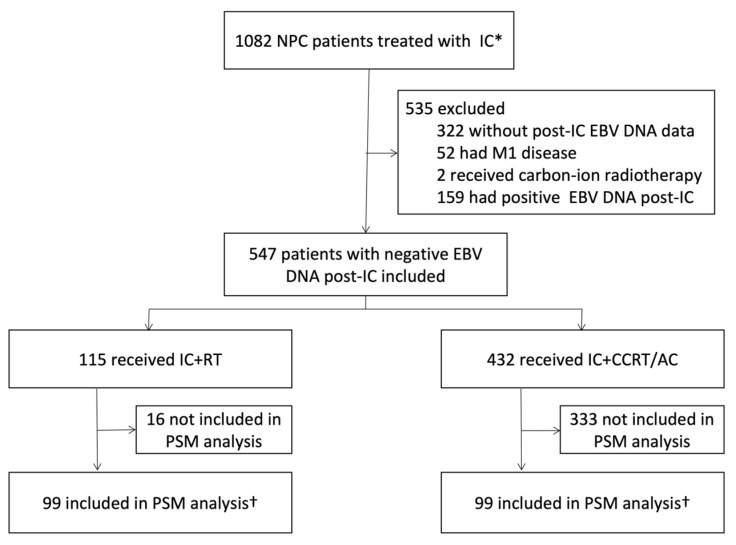
Flow Diagram of Enrollment. * Started receiving induction chemotherapy (IC) between September 2017 and November 2020. ^†^ Propensity score matching by gender, age, T category, N category, clinical stage, pre-IC EBV DNA levels, IC regimen, IC cycle, and targeted therapy. Abbreviations: NPC, nasopharyngeal carcinoma; IC, induction chemotherapy; EBV DNA, Epstein-Barr virus (EBV) DNA; RT, radiotherapy; CCRT, concurrent chemoradiotherapy; AC, adjuvant chemotherapy; PSM, propensity score matching.

NPC patients treated with IC at our Center were retrospectively reviewed between September 2017 and November 2020. The inclusion criteria were as follows: (1) treatment-naive, pathologically confirmed NPC patients; (2) no evidence of distant metastasis; (3) treated with IC before radical IMRT; (4) negative EBV DNA level (<500 copies/mL) after IC; and (5) without previous or concomitant malignancies. A total of 547 patients who met the criteria were included in this study (Figure 1).

All patients were evaluated by detailed medical history, physical examination, magnetic resonance imaging (MRI) (preferred) or computed tomography (CT) of the head and neck, chest CT, bone scan, abdominal ultrasonography or whole-body fluorodeoxyglucose positron emission tomography CT (PET/CT), fiberoptic nasopharyngoscopy or indirect nasopharyngoscopy, electrocardiogram, and complete blood sampling, including plasma EBV DNA level, before the start of treatment. All patients were staged according to the 8th edition of the International Union Against Cancer/American Joint Committee on Cancer (UICC/AJCC) staging system. This study was approved by the Institutional Review Board of our Cancer Center (No. 2009224-1).

### 2.2. Plasma EBV DNA Detection

Peripheral venous blood (5 mL) was obtained for plasma EBV DNA detection using real-time quantitative polymerase chain reaction (qPCR). Post-IC EBV DNA was detected within one week before the start of IMRT. The EBV DNA levels were defined as positive (≥500 copies/mL) or negative (<500 copies/mL), according to the standards of our center.

### 2.3. Treatments

All patients underwent cisplatin-based IC, including TP (docetaxel 60–75 mg/m^2^ day 1, cisplatin 75 mg/m^2^ day 1 or 25 mg/m^2^/day days 1–3), GP (gemcitabine 1000 mg/m^2^ day 1 and day 8, cisplatin 75 mg/m^2^ day 1 or 25 mg/m^2^/day days 1–3), or PF (cisplatin 25 mg/m^2^/day days 1–3, 5-fluorouracil 500 mg/m^2^/day, continuous intravenous infusion for 120 h). The IC was administered every three weeks for 2 or 3 cycles. This was followed by IMRT, with or without concurrent cisplatin (30–40 mg/m^2^ weekly or 75–80 mg/m^2^ every three weeks), or adjuvant chemotherapy. The AC regimen was the same as the IC and was started one month after IMRT.

IMRT was delivered following our institutional treatment protocol. The target volume was defined according to the International Commission on Radiation Units (ICRU) and Measurements Reports 59 and 62 [19]. The gross tumor volume (GTV) was based on the post-IC images for intracavity tumors and lymph nodes. For infiltration tumors (bony structures of the skull base and cervical vertebra invasion) and extracellular invasion of lymph nodes, the GTV was based on the pre-IC images. The prescribed dose was 66–70.4 Gy to GTV (including the primary tumor and positive lymph nodes), 60 Gy to CTV1 (high-risk clinical target volume), and 54 Gy to CTV2 (low-risk clinical target volume) in 30–35 fractions. The simultaneous integrated boost (SIB) technique was used for all of the target volumes. More details have been described in a previous study [20].

### 2.4. Follow-Up and Assessment

Patients were followed up weekly during IMRT. After treatment, patients were assessed every three months in the first two years, every six months in the third to fifth years, and then annually thereafter.

Follow-up assessments included examination of the head and neck, EBV DNA levels, and thyroid and pituitary function tests. MRI of the nasopharynx, chest CT scan, and ultrasound or CT of the neck and abdomen were performed every 6–12 months. Additional tests were recommended when clinically indicated. Acute toxicities were graded according to the National Cancer Institute Common Toxicity Criteria for Adverse Events (version 4.02). The locoregional relapse-free survival (LRFS), distant metastasis-free survival (DMFS), overall survival (OS), and progression-free survival (PFS) rates were calculated from the day of the first treatment to the day of each event occurred or the last follow-up.

### 2.5. Statistical Analysis

The χ^2^ test or Fisher’s exact test was used to compare clinicopathologic and treatment characteristics between groups. The LRFS, DMFS, OS, and PFS rates were estimated and compared using the Kaplan–Meier method and log-rank tests. The Cox regression model was used for multivariate analyses. Patients were classified into the IC + RT group and the IC + CCRT/AC group. The IC + CCRT/AC group included patients who were treated with IC + CCRT and IC + RT + AC. One-to-one propensity score matching was performed using logistic regression, with a nearest-neighbor caliper width of 0.1 to optimize the comparability of different treatment groups. Covariates used for matching included gender, age, T category, N category, clinical stage, pre-IC EBV DNA levels, IC regimen, IC cycle, and targeted therapy. All the statistical analysis was performed using the Statistical Package for Social Sciences (SPSS version 20.0) and R (version 4.2.2). Two-sided *p* values < 0.05 were considered statistically significant.

## 3. Results

### 3.1. Patient and Treatment Characteristics

The baseline characteristics of the 547 patients are summarized in Table 1. Among them, 115 patients received IC + RT, and 432 received IC + CCRT/AC. Before PSM, patients treated with IC + CCRT/AC tended to be younger (<45 years, *p* = 0.002) and have more advanced diseases (N2–3, *p* = 0.023; stage IVa, *p* = 0.007) than those with IC + RT. IC regimen and cycle were also unbalanced, with more patients receiving a GP regimen in the IC + CCRT/AC group (*p* < 0.001), and most of them received 2 cycles (*p* < 0.001). There was no significant difference in gender, T category, pre-IC EBV DNA levels, and targeted therapy. After one-to-one propensity score matching, 99 patients from each group were enrolled in the study. All variables were well-balanced between the two groups (Table 1).

In the IC + CCRT/AC group, a total of 205 patients received AC, of which 147 patients (71.7%) completed 2 cycles, 6 patients (2.9%) completed 3 cycles, and 52 patients (25.4%) completed only 1 cycle due to intolerable toxicities or refusal by patients. A total of 227 patients received concurrent chemotherapy. For patients who received a weekly cisplatin regimen (*n* = 158), 62 (39.2%) patients received only 1–3 cycles, and 96 (60.8%) patients completed 4–7 cycles. For patients who received every 3 weeks a cisplatin regimen (*n* = 69), 27 patients (39.1%) completed only 1 cycle, and 42 patients (60.9%) completed 2–3 cycles. The median total dose of concurrent cisplatin was 120 mg/m^2^ (IQR 90–150).

### 3.2. Survival Outcomes

With a median follow-up time of 37 months (range, 8–58 months) for the original cohort and 38 months (range, 12–58 months) for the PSM cohort, the estimated 3-year LRFS, DMFS, OS, and PFS were 92.2%, 92.4%, 96.4%, and 84.4% in the original cohort and 91.8%, 95.3%, 94.2%, and 85.2% in the PSM cohort, respectively.

**Figure 2:**
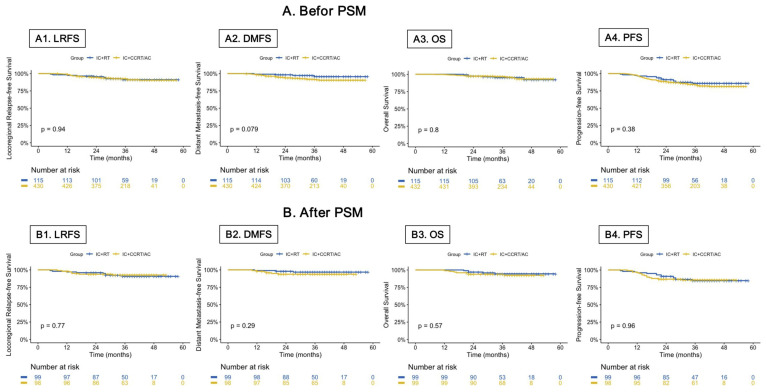
Survival comparisons between IC + RT and IC + CCRT/AC groups before propensity score matching (**A**) and after propensity score matching (**B**), including LRFS (1), DMFS (2), OS (3), and PFS (4). Abbreviations: PSM, propensity score matching; LRFS, locoregional relapse-free survival; DMFS, distant metastasis-free survival; OS, overall survival; PFS, progression-free survival; IC, induction chemotherapy; RT, radiotherapy; CCRT, concurrent chemoradiotherapy; AC, adjuvant chemotherapy.

There was no significant difference in LRFS, DMFS, OS, and PFS between the IC + RT and the IC + CCRT/AC group in the original cohort. The 3-year LRFS, DMFS, OS, and PFS rates were 91.1% vs. 92.6% (IC + RT group vs. IC + CCRT/AC group, *p* = 0.94), 95.6% vs. 91.5% (*p* = 0.08), 95.2% vs. 96.8% (*p* = 0.80), and 85.9% vs. 84.0%, (*p* = 0.38), respectively (Figure 2(A1–A4)). The same result was observed in the PSM cohort. Patients treated with IC + RT had similar survival rates compared to those with IC + CCRT/AC. The 3-year LRFS, DMFS, OS, and PFS rates were 90.7% vs. 92.7% (*p* = 0.77), 96.8% vs. 93.7%, (*p* = 0.29), 94.5% vs. 93.9% (*p* = 0.57), and 84.7% vs. 85.6% (*p* = 0.96), respectively (Figure 2(B1–B4)).

### 3.3. Prognostic Analysis

We did univariate and multivariate analyses for LRFS, DMFS, OS, and PFS in the original cohort. The results demonstrated that the treatment schedule was not an independent predictor for all survival rates. In contrast, the clinical stage was an independent predictor for LRFS (hazard ratio, 1.97; 95% confidence interval, 1.06–3.67; *p* = 0.03), DMFS (hazard ratio, 2.48; 95% confidence interval, 1.26–4.85; *p* < 0.01), OS (hazard ratio, 4.56; 95% confidence interval, 1.78–11.63; *p* < 0.01), and PFS (hazard ratio, 2.06; 95% confidence interval, 1.28–3.31; *p* < 0.01). Patients with stage IVa disease had significantly inferior survival rates than those with stage II–III. We also found that the GP regimen was associated with superior OS (hazard ratio, 0.27; 95% confidence interval, 0.11–0.70; *p* < 0.01) than the TP or PF regimen. The univariate analysis showed that patients with pre-IC EBV DNA < 500 copies/mL had better DMFS than those with pre-IC EBV DNA ≥ 500 copies/mL. However, the multivariate analysis showed that pre-IC EBV DNA was not an independent predictor for DMFS (hazard ratio, 0.44; 95% confidence interval, 0.17–1.13; *p* = 0.09). Details are shown in Tables S1 and S2.

### 3.4. Treatment Failures

Patterns of treatment failure were analyzed in the original cohort. At the time of the last follow-up, a total of 34 (7.9%) patients in the IC + CCRT/AC and 9 (7.8%) in the IC + RT group had locoregional recurrences. Distant failure was found in 35 (8.1%) and 4 (3.5%) patients in the IC + CCRT/AC and IC + RT groups, respectively. Twenty-five patients died. Cancer-specific death accounted for 64% of all death. Details are shown in Table 2.

**Figure 3:**
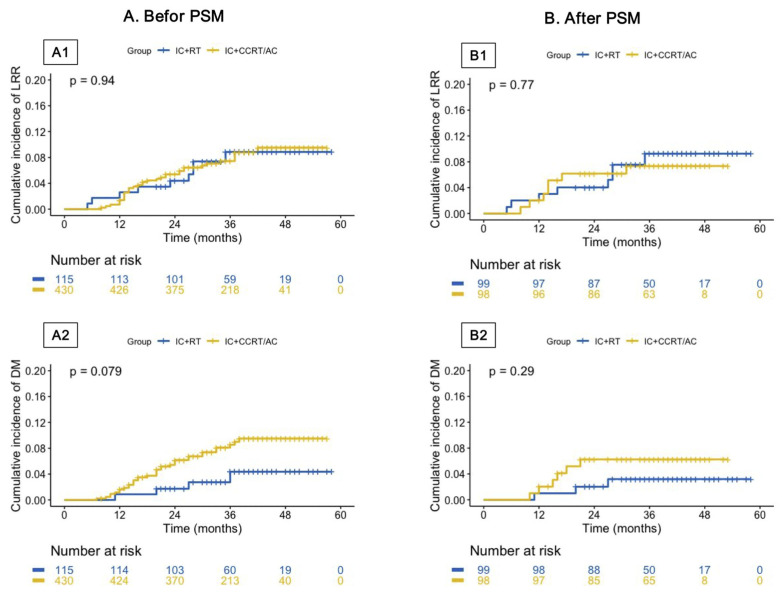
Cumulative incidence of locoregional relapse (LRR, (**A1**,**B1**)) and distant metastasis (DM, (**A2**,**B2**)) among patients who underwent IC + RT and IC + CCRT/AC. (**A**) Before propensity score matching analysis. (**B**) After propensity score matching analysis. Abbreviations: PSM, propensity score matching; LRR, locoregional relapse; DMFS, distant metastasis; IC, induction chemotherapy; RT, radiotherapy; CCRT, concurrent chemoradiotherapy; AC, adjuvant chemotherapy.

There was no significant difference in the cumulative incidence of locoregional relapse (LRR) and distant metastasis (DM) between the two groups. In the original cohort, the 3-year cumulative incidence of LRR and DM among patients who underwent IC + CCRT/AC and IC + RT was 7.9% vs. 7.8% (*p* = 0.977) and 8.1% vs. 3.5% (*p* = 0.085), respectively (Figure 3A). In the PSM cohort, the 3-year cumulative incidence of LRR and DM for patients treated with IC + CCRT/AC and IC + RT was 7.1% vs. 8.1% (*p* = 0.804) and 6.1% vs. 3.0% (*p* = 0.485), respectively (Figure 3B).

### 3.5. Toxicities

Treatment-related acute toxicities for different treatment groups in the PSM cohort were evaluated. Grade 3–4 hematological toxicities were more frequently observed in the IC + CCRT group than in the IC + RT group. Patients in the IC + CCRT group were more likely to suffer from grade 3–4 leukocytopenia (13.6 vs. 2%, *p* = 0.017) and anemia (6.8 vs. 0%, *p* = 0.046), as compared with those in the IC + RT alone group. There was no significant difference in grade 3–4 dermatitis and mucositis between the two groups. However, grade 2 mucositis was more frequently observed in the IC + CCRT group than in the IC + RT group (43.2% vs. 24.2%, *p* = 0.023). Treatment-related toxicities are shown in Table 3.

## 4. Discussion

In this study, we found that IC + RT displayed similar survival outcomes as IC + CCRT/AC for NPC patients with negative post-IC EBV DNA. The omission of concurrent or adjuvant chemotherapy did not increase locoregional or distant failure. However, patients treated with IC + RT had fewer acute toxicities than those with IC + CCRT/AC. The data were significant because this is the first study to evaluate de-intensified treatment in patients with negative post-IC EBV DNA. Our results indicated the feasibility of omitting CCT and AC for NPC patients with negative post-IC EBV DNA.

IC + CCRT has been recommended as the standard treatment for LA-NPC since 2020 [2]. However, CCT was associated with increased toxicities, poor tolerance, and low completion rates [4,5,6,7,21,22]. In the study by Zhang et al. [22], grade 3–4 toxicities happened in 75.7% of patients treated with CCRT, and only 38.9% of patients completed the established 3 cycles of CCT. Recently, several retrospective studies explored the de-intensified treatment of omitting CCT after IC, but the results were inconsistent [10,11,12,13]. In the study by He et al. [11], the toxicities and survival rates were not different in IC + RT and CCRT groups. However, Wang et al. [10] found that the omission of CCT was associated with worse PFS. It is worth noting that neither of these studies made risk stratification for patients. In the study by Liu et al. [12], patients were stratified into three risk groups according to the nomogram scores (including age, gender, TNM stage, and baseline EBV DNA). For low- and medium-risk patients, IC + RT and CCRT showed similar efficacy. However, for high-risk patients, IC + RT displayed inferior 5-year OS (71.0% vs. 77.2%, *p* = 0.022) and PFS (69.4% vs. 75.4%, *p* = 0.019) compared with CCRT. However, the complexity of the nomogram limited its clinical application. This highlights the need for establishing handy and effective methods for risk stratification to guide individualized clinical treatment.

In this study, post-IC EBV DNA was selected as the only risk stratification factor because compared with the traditional TNM stage or baseline EBV DNA, post-IC EBV DNA was dynamic and reflected the chemosensitivity of the tumor. An increasing number of studies have confirmed the prognostic role of post-IC EBV DNA [15,16,17,18]. Chen et al. [18] retrospectively analyzed 910 LA-NPC patients and found that negative post-IC EBV DNA was associated with superior 5-year OS, DMFS, and DFS. Similar results have been reported in other studies [16,17,23]. All of the above indicated the potential value of post-IC EBV DNA in risk stratification. Hence, in the present study, we used the post-IC EBV DNA level as a risk stratification factor, and patients with negative post-IC EBV DNA were defined as low-risk. We found no significant difference in LRFS, DMFS, OS, and PFS in the original cohort between the IC + RT and IC + CCRT/AC groups. The results remained consistent in the PSM cohort after balancing the baseline characteristics (including gender, age, stage, baseline EBV DNA, IC regen, IC cycle, and target therapy). However, patients in the IC + RT group had fewer treatment-related acute toxicities and better tolerance.

The Cox regression analysis showed that the treatment schedule was not an independent prognostic factor for LRFS, DMFS, OS, and PFS. There was no significant difference in the cumulative incidence of LRR and DM between the IC + RT and IC + CCRT/AC groups, which meant that omitting concurrent or adjuvant chemotherapy did not increase locoregional or distant failure in this low-risk population. Prospective clinical trials are needed to further confirm our findings.

Regarding the treatment-related toxicities, we found that Grade 3–4 hematological toxicities and Grade 2 mucositis occurred more frequently in the IC + CCRT group than in the IC + RT group. We attributed that to the bone marrow suppression caused by concurrent chemotherapy and the gastrointestinal toxicities caused by concurrent chemoradiotherapy, including nausea, vomiting, diarrhea, and mucositis, which caused inadequate nutrition intake. Furthermore, the chemotherapy completion rate was unsatisfactory in the IC + CCRT/AC group. About 39% of the patients received only 1 cycle of concurrent chemotherapy, and 25.4% received only 1 cycle of adjuvant chemotherapy due to intolerable toxicities or refusal by patients. The completion rate of concurrent chemotherapy in this study was lower than that reported in the literature (87.3–92%) [21,22]. Possible reasons included different IC regimens and older patients. The median age in our study was 49 years (range 13–76 years) and 42–46 years (range 18–64 years) in the literature. In addition, 14.8% of patients were older than 60 in our study. In terms of AC, the completion rate was 71.7% in our study. In the phase 3 clinical trial of Chen et al. [24] aiming to compare the effect of CCRT + AC and CCRT alone, the completion rate of AC in the CCRT + AC group was 63%. It should be noted that the protocol of the AC regimen was three cycles in Chen’s study [24], while in our study, two cycles of AC were mostly used. So, it cannot be directly compared due to the different treatment protocols. However, it was certain that the tolerance of concurrent chemotherapy after IC and AC after CCRT/RT was generally poor. In the phase 3 clinical trial of Sun et al. [25] comparing TPF induction chemotherapy plus concurrent chemoradiotherapy (IC + CCRT) with CCRT alone, the completion rate of concurrent chemotherapy in the IC + CCRT group was significantly lower than that in the CCRT group (30% vs. 56%). Similarly, in the study by Zhang et al. [22], the completion rates of concurrent chemotherapy in the IC + CCRT group and the CCRT group were 38.9% and 74.7%, respectively.

It is worthwhile to note that the AC regimens in our study were relatively high-intensity regimens, such as TP, GP, and PF. The poor tolerance of patients may affect the efficacy. Chen et al. [26] published the results of a phase 3 clinical trial of metronomic capecitabine as adjuvant therapy in locoregionally advanced NPC in 2021, which showed that the addition of metronomic adjuvant capecitabine to CCRT significantly improved the failure-free survival (FFS) rate in patients with high-risk locoregionally advanced NPC (with a 3-year FFS of 85.3% in the CCRT + AC group and 75.7% in the CCRT group, *p* = 0.0023), while the toxicities were manageable, and there was no compromise to the quality of life. With the emergence of new low-toxicity and effective adjuvant therapy (capecitabine, immunotherapy, etc.), the efficacy of induction chemotherapy combined with adjuvant chemotherapy deserves further exploration.

There were several limitations in our study. First, this was a retrospective study from a single center. Even though we did PSM analysis, patient selection and treatment assignment bias could not be avoided. Second, late toxicities were not reported due to the limited follow-up time, and a longer follow-up duration is required. Third, the lack of quality-of-life data may make the results underpowered. Despite these limitations, our study provides the only evidence to date that LRFS, DMFS, OS, and PFS are not different from IC + RT vs. IC + CCRT/AC in patients with negative post-IC EBV DNA.

## 5. Conclusions

In summary, our study showed that IC + RT displayed similar survival outcomes as IC + CCRT/AC for NPC patients with negative post-IC EBV DNA. The omission of concurrent or adjuvant chemotherapy did not increase locoregional or distant failure, but the toxicities were significantly reduced. Prospective clinical trials are needed to further confirm our findings.

## Figures and Tables

**Table 1:** Patient characteristics of the original and the PSM cohorts.

Characteristics		Before PSM (*n* = 547)			After PSM (*n* = 198)		
		IC + CCRT/AC (*n* = 432) *n* (%)	IC + RT (*n* = 115) *n* (%)	*p* Value	IC + CCRT/AC (*n* = 99) *n* (%)	IC + RT (*n* = 99) *n* (%)	*p* Value
Age (years)	<45	168 (38.9)	26 (22.6)	0.002	30 (30.3)	26 (26.3)	0.636
	≥45	264 (61.1)	89 (77.4)		69 (69.7)	73 (73.7)	
Gender	Female	118 (27.3)	36 (31.3)	0.466	27 (27.3)	28 (28.3)	1.000
	Male	314 (72.7)	79 (68.7)		72 (72.7)	71 (71.7)	
T category ^a^	T1–2	129 (29.9)	34 (29.6)	1.000	30 (30.3)	32 (32.3)	0.878
	T3–4	303 (70.1)	81 (70.4)		69 (69.7)	67 (67.7)	
N category ^a^	N0–1	116 (26.9)	44 (38.3)	0.023	28 (28.3)	35 (35.4)	0.360
	N2–3	316 (73.1)	71 (61.7)		71 (71.7)	64 (64.6)	
Clinical stage ^a^	II-III	215 (49.8)	74 (64.3)	0.007	57 (57.6)	58 (58.6)	1.000
	IVa	217 (50.2)	41 (35.7)		42 (42.4)	41 (41.4)	
Pre-IC EBV DNA (copies/mL)	<500	120 (27.8)	39 (33.9)	0.241	31 (31.3)	33 (33.3)	0.879
	≥500	312 (72.2)	76 (66.1)		68 (68.7)	66 (66.7)	
IC regimen	TP	196 (45.4)	91 (79.1)	<0.001	77 (77.8)	75 (75.8)	0.127
	GP	232 (53.7)	20 (17.4)		22 (22.2)	20 (20.2)	
	PF	4 (0.9)	4 (3.5)		0 (0.0)	4 (4.0)	
IC cycle	1	5 (1.2)	3 (2.6)	<0.001	1 (1.0)	3 (3.0)	0.551
	2	317 (73.4)	58 (50.4)		57 (57.6)	53 (53.5)	
	≥3	110 (25.5)	54 (47.0)		41 (41.4)	43 (43.4)	
Targeted therapy ^b^	no	371 (85.9)	100 (87.0)	0.885	84 (84.8)	84 (84.8)	1.000
	yes	61 (14.1)	15 (13.0)		15 (15.2)	15 (15.2)	

Abbreviation: PSM, propensity score matching; IC, induction chemotherapy; CCRT, concurrent chemoradiotherapy; AC, adjuvant chemotherapy; RT, radiotherapy; EBV DNA, Epstein–Barr virus (EBV) DNA. ^a^ According to the 8th edition of the International Union Against Cancer/American Joint Committee on Cancer (UICC/AJCC) staging system. ^b^ Nimotuzumab.

**Table 2:** Patterns of treatment failure.

Site	IC + CCRT/AC (*n* = 432) *n* (%)	IC + RT (*n* = 115) *n* (%)
Locoregional recurrence		
Local only	20 (4.6)	3 (2.6)
Regional only	4 (0.9)	4 (3.5)
Local and regional	4 (0.9)	1 (0.9)
Distant metastases		
Distant only	29 (6.7)	3 (2.6)
Local and distant	1 (0.2)	1 (0.9)
Regional and distant	3 (0.7)	0
Local, regional, and distant	2 (0.5)	0
Total locoregional failure	34 (7.9)	9 (7.8)
Total distant failure	35 (8.1)	4 (3.5)
Death (any cause)	19 (4.4)	6 (5.2)
NPC cause	14 (3.2)	2 (1.7)
Non-NPC cause	0	1 (0.9)
Unknow cause	5 (1.2)	3 (2.6)

Abbreviation: IC, induction chemotherapy; CCRT, concurrent chemoradiotherapy; AC, adjuvant chemotherapy; RT, radiotherapy; NPC, nasopharyngeal carcinoma.

**Table 3:** Incidences of grade 3–4 treatment-related toxicities for the PSM cohort.

Toxicity	IC + CCRT (*n* = 44) *n* (%)	IC + RT (*n* = 99) *n* (%)	*p* Value
Leukopenia	6 (13.6)	2 (2)	0.017
Neutropenia	0	1 (1)	1.000
Anemia	3 (6.8)	0	0.046
Thrombocytopenia	1 (2.3)	0	0.676
Mucositis	17 (38.6)	37 (37.4)	0.886
Dermatitis	5 (11.4)	9 (9.1)	0.907
AST/ALT increase	0	0	-
BUN increase	0	0	-
Creatinine increase	0	0	-

Abbreviation: IC, induction chemotherapy; CCRT, concurrent chemoradiotherapy; RT, radiotherapy; AST, aspartate aminotransferase; ALT, alanine aminotransferase; BUN, blood urea nitrogen.

## Data Availability

The datasets used and/or analyzed during the current study are available from the corresponding author upon reasonable request.

## Supplementary Materials

The following supporting information can be downloaded at: https://www.mdpi.com/article/10.3390/cancers15061689/s1, Table S1. Impact of prognostic factors on treatment results by univariate analysis in 547 patients with negative EBV DAN post-induction chemotherapy. Table S2. Cox multivariate regression analyses for predictors of survival in 547 patients with negative EBV DAN post-induction chemotherapy.
