# Supplementary material for: Radiotherapy Alone Versus Concurrent or Adjuvant Chemoradiotherapy for Nasopharyngeal Carcinoma Patients with Negative Epstein–Barr Virus DNA after Induction Chemotherapy
Source: Cancers (Basel). 2023 Mar 9;15(6):1689. doi: 10.3390/cancers15061689 (PMC10046756; doi:10.3390/cancers15061689)
Supplement: Supplementary file 1 [file cancers-15-01689-s001.zip › cancers-2215683-supplementary.pdf]

Table S1. Impact of prognostic factors on treatment results by univariate analysis in 547 patients with negative EBV DAN post-induction chemotherapy.

Items	p value			
	LRFS	DMFS	OS	PFS
Age (yr)				
<45 vs. ≥45	0.068	0.942	0.036	0.282
Gender				
Male vs. Female	0.463	0.288	0.339	0.608
T category (AJCC 8th)				
T1-2 vs. T3-4	0.277	0.150	0.232	0.064
N category (AJCC 8th)				
N0-1 vs. N2-3	0.426	0.351	0.806	0.762
Clinical stage (AJCC 8th)				
Stage II-III vs. Stage IVa	0.024	0.009	0.003	0.001
Pre-IC EBV DNA (copies/mL)				
<500 vs. ≥500	0.886	0.025	0.353	0.211
IC regimen				
TP vs. GP vs. PF	0.604	0.180	0.080	0.439
IC cycle				
1 vs. 2 vs. ≥3	0.227	0.496	0.126	0.331
Targeted therapy				
no vs. yes	0.045	0.400	0.833	0.255
Treatment schedule				
IC+RT vs. IC+CCRT/AC	0.936	0.079	0.797	0.352

Abbreviation: LRFS, locoregional relapse-free survival; DMFS, distant metastasis-free survival; OS, overall survival; PFS, progression-free survival; AJCC = American Joint Committee on Cancer; IC, induction chemotherapy; EBV DNA, Epstein-Barr virus (EBV) DNA; TP, docetaxel plus cisplatin; GP, gemcitabine plus cisplatin; PF, cisplatin plus 5-fluorouracil; RT, radiotherapy; CCRT, concurrent chemoradiotherapy; AC, adjuvant chemotherapy.

Table S2. Cox multivariate regression analyses for predictors of survival in 547 patients with negative EBV DAN post-induction chemotherapy.

Variable	Hazard ratio	95% CI	p value
LRFS			
Clinical stage (IVa vs. II-II)	1.972	1.059-3.673	0.032
DMFS			
Clinical stage (IVa vs. II-II)	2.476	1.262-4.854	0.008
OS			
Clinical stage (IVa vs. II-II)	4.555	1.784-11.632	0.002
IC regimen (GP vs.TP)	0.272	0.105-0.700	0.007
IC cycle (≥3 vs. 1)	0.083	0.009-0.783	0.030
PFS			
Clinical stage (IVa vs. II-II)	2.060	1.284-3.306	0.003

Abbreviation: EBV DNA, Epstein-Barr virus (EBV) DNA; CI = confidence interval; LRFS, locoregional relapse-free survival; DMFS, distant metastasis-free survival; OS, overall survival; PFS, progression-free survival; IC, induction chemotherapy; GP, gemcitabine plus cisplatin; TP, docetaxel plus cisplatin.
